# Case Report: Locally invasive thyroid metastases from renal cell carcinoma: surgery after neoadjuvant therapy

**DOI:** 10.3389/fonc.2025.1543060

**Published:** 2025-05-28

**Authors:** Dana M. Hartl, Mohamed-Amine Bani, Abir Al Ghuzlan, Andreea-Elena Simonescu, Ingrid Breuskin, Alix Marhic, Laurence Albiges, Livia Lamartina, Julien Hadoux

**Affiliations:** ^1^ Division of Surgery and Anesthesiology, Head and Neck Oncology Service, Thyroid Surgery Unit, Gustave Roussy Cancer Campus Grand Paris, Villejuif, France; ^2^ Department of Pathology, Gustave Roussy Cancer Campus, Villejuif, France; ^3^ Department of Medical Oncology, Gustave Roussy Cancer Campus, Villejuif, France; ^4^ University of Paris-Saclay, Le Kremlin-Bicêtre, France; ^5^ Department of Medical Imaging, Endocrine Oncology Service, Gustave Roussy Cancer Campus, Villejuif, France; ^6^ Endocan-Tuthyref Network, Villejuif, France

**Keywords:** renal cell carcinoma, thyroid, metastasis, neoadjuvant therapy, kinase inhibitors, immunotherapy, surgery

## Abstract

**Objective:**

Neoadjuvant therapy is under investigation not only for unresectable clear cell renal cell carcinoma (ccRCC) (1) but also for locally invasive primary thyroid cancers (2). Herein, we describe two cases of locally invasive thyroid metastases from ccRCC treated surgically after neoadjuvant therapy to highlight the rationale and outcomes.

**Patients and methods:**

Two patients, one woman and one man, both age 69, developed unresectable thyroid metastases from ccRCC, respectively, 20 and 13 years after nephrectomy for ccRCC. Patient 1 received lenvatinib and a bispecific anti–Programmed cell Death protein 1/ cytotoxic T-lymphocyte-associated protein 4 (PD-1/CTLA-4) antibody in the context of a clinical trial. The second patient received nivolumab and cabozantinib.

**Results:**

The observed tumor response in patient 1 showed a decrease in mean surgical complexity score from unresectable (prevertebral fascia invasion) to severe (risk of recurrent nerve paralysis) and in patient 2 from unresectable (prevertebral fascia) to moderate (superficial esophageal invasion). The recurrent nerve was invaded in patient 1, leading to a subtotal resection. Surgery was a total thyroidectomy extended to the internal jugular vein in patient 2. Hospitalization was 1 and 2 days, respectively. Postoperative dysphonia improved in patient 1 after 3 months. No complications occurred in the second patient, who received adjuvant radiation therapy. After surgery, systemic therapy was discontinued in both patients, and stable residual oligometastatic disease was followed.

**Conclusion:**

Neoadjuvant therapy enabled a macroscopic resection of locally invasive thyroid metastases, preserving laryngeal function and allowing discontinuation of systemic therapy. This approach may be considered in these rare cases, although the impact on progression-free or overall survival is currently unknown.

## Introduction

Metastases to the thyroid gland can occur in any type of metastatic tumor, with an estimated incidence from autopsy series of 2%. The most common primary tumor metastizing to the thyroid on autopsy is lung cancer, but, in clinically detected thyroid metastases, clear cell renal cell carcinoma (ccRCC) is the most frequent primary tumor, representing an estimated 25% of all thyroid metastases (3). Up to 90% of these metastases are metachronous, occurring years or decades after the initial nephrectomy (4).

For locally unresectable ccRCC, neoadjuvant therapy may be offered in the context of a clinical trial, but there is currently no high-level evidence showing improved survival in patients receiving neoadjuvant therapy for their primary tumor (1, 5). High rates of response have been reported in patients with combinations of tyrosine kinase inhibitors and/or immune checkpoint inhibitors such as nivolumab plus ipilimumab, pembrolizumab plus axitinib, lenvatinib plus pembrolizumab or nivolumab, and cabozantinib. Likewise, there has recently been a paradigm shift for locally invasive primary thyroid malignancies, with complete (R0/R1) resection being attained after neoadjuvant treatment with tyrosine kinase inhibitors (6).

For oligometastatic disease, the 2024 European Association of Urology guidelines state that, in an attempt to control local symptoms, ablative therapy, including metastasectomy, may be proposed to patients with metastatic disease and favorable disease factors and in whom complete resection is achievable (weak recommendation). In many patients, distant metastases, particularly in the lung and pancreas, can spontaneously remain stable over many years. Most reported thyroid metastases are strictly intrathyroidal and amenable to standard thyroid surgery, if symptomatic (7). There are few reports, however, of locally invasive unresectable thyroid metastases treated with systemic therapy and subsequently resected (8).

We report two cases of thyroid metastases from ccRCC in oligometastatic patients that were initially judged to be unresectable but then became amenable to surgical resection after response to systemic therapy with tyrosine kinase inhibitors and immunotherapy. The clinical and histopathological characteristics of these tumor responses and patient outcomes are described.

## Patients and methods

**Figure 1:**
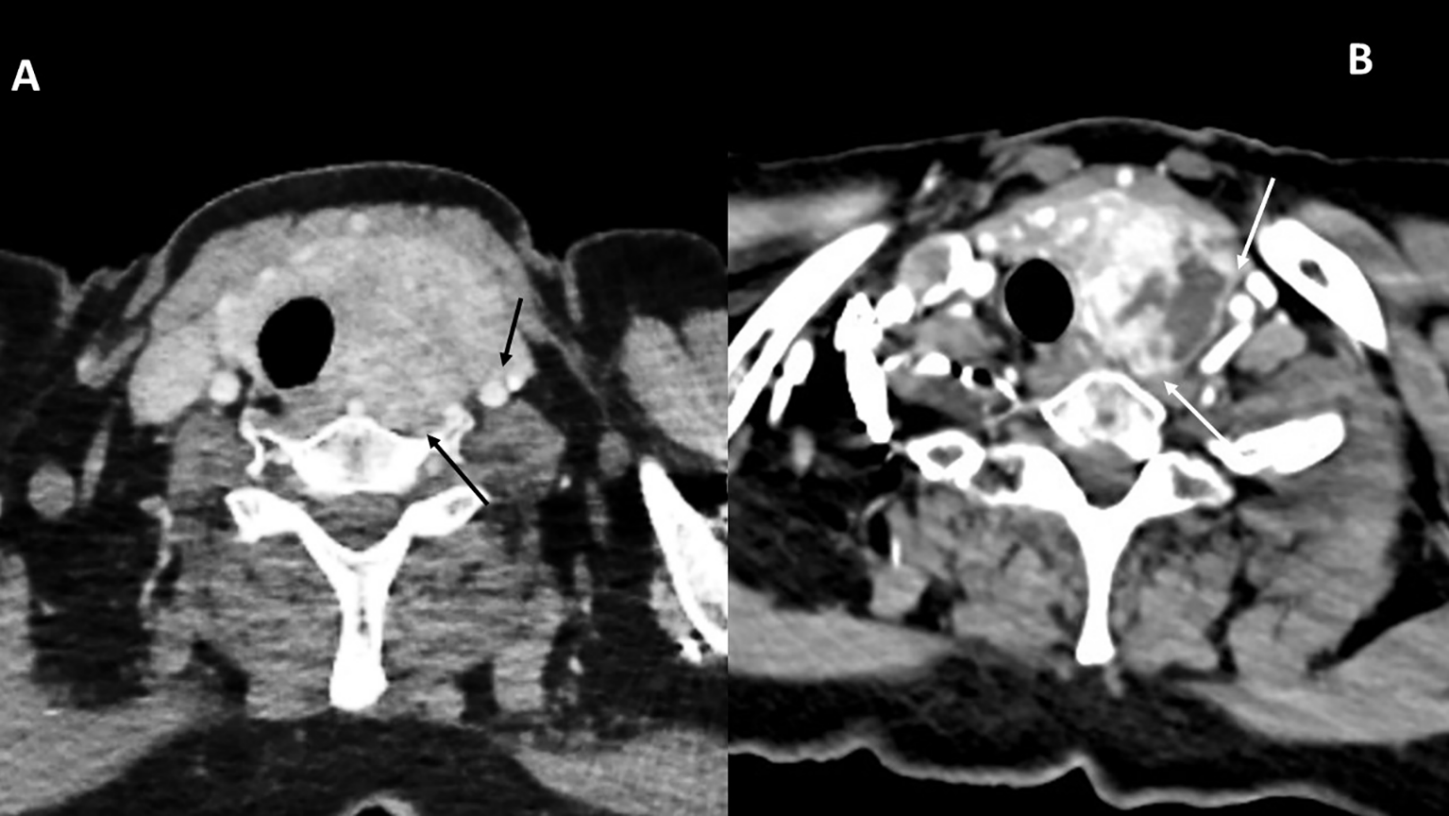
CT scan images for patient 1, before systemic therapy **(A)** and 3 months after discontinuation of both drugs **(B)**. The involvement of the prevertebral fascia and common carotid artery classified this tumor as initially unresectable (black arrows, **A**). Tumor response is indicated in **B** (white arrows).

Patient 1 was a 69-year-old woman who had been treated with a radical nephrectomy 20 years previously for ccRCC stage T2N0M0. During the follow-up, 14 years later, she was diagnosed with a multiorgan relapse, involving contralateral kidney, pancreas, thyroid, and a single-level V later cervical lymph node. The contralateral ccRCC was treated by tumorectomy, Radiation therapy was administered to the neck node, due to symptoms and to avoid surgery due to the proximity of the spinal accessory nerve. The left thyroid lobe metastasis remained under active surveillance for 6 years. Local progression led to the initiation of systemic first-line therapy with lenvatinib plus a bispecific anti–PD-1 and anti–CTLA-4 antibody in the context of a clinical trial. Vocal fold mobility was normal. At initiation of systemic therapy, the thyroid metastasis was judged unresectable due to involvement of the esophageal muscle, the common carotid artery, and the prevertebral fascia (black arrows, **Figure 1**).

**Figure 2:**
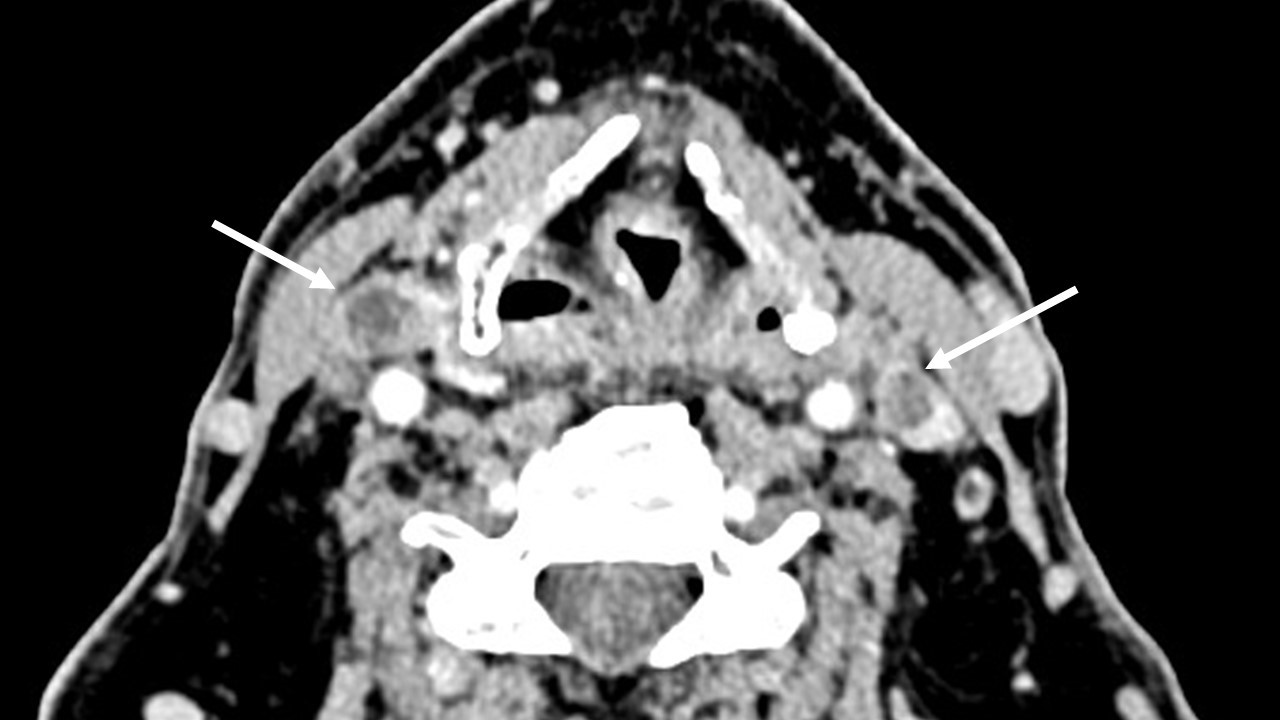
Tumor thrombus in both internal jugular veins in patient 2 before therapy (white arrows).

Patient 2 was a 69-year-old man who had undergone a radical nephrectomy 14 years earlier at an outside institution for a T1Nx clear cell renal carcinoma (grade not specified) and then two other surgical procedures for a local recurrence and for retroperitoneal adenopathy 9 years earlier (grade not specified). He was progression-free with no treatment for 9 years when he was diagnosed with multinodular goiter. Vocal fold mobility was normal. Surgical resection was attempted at an outside institution, but the tumor was deemed to be unresectable. fluorodeoxyglucose (FDG)-positron emission tomography (PET) (^18^FDG-PET), performed because of the suspicion of anaplastic thyroid carcinoma, revealed uptake in the right adrenal gland, with no tumor otherwise visible, and uptake in a small lung nodule. On computed tomography, the subglottic trachea was massively invaded. Tumor thrombi were observed in both internal jugular veins (**Figure 2**). The biopsy revealed a thyroid metastasis from ccRCC. The patient was deemed oligometastatic, but, due to the tracheal invasion with a risk of severe respiratory complications, it was decided to start systemic therapy with cabozantinib and nivolumab.

## Results

### Patient 1

Systemic therapy was poorly tolerated with grade 3 hepatic toxicity and fever, leading to discontinuation after a total of 4 weeks of treatment. A clinical response with a decrease in the volume of the thyroid metastasis was nonetheless observed. At imaging 3 months later, the tumor showed shrinkage in the region of the great vessels and the prevertebral fascia. In terms of the Thyroid Neck Group Morbidity Complexity Score (**Table 1**), the tumor regressed from class 4—unresectable—to class 2—risk for unilateral recurrent laryngeal nerve paralysis (2, 6). In terms of the Massachusetts General Hospital/Mass Eye and Ear–Memorial Sloan Kettering–MD Anderson (MMM) surgical morbidity complexity score, the lesion regressed from a score of 18 to a score of 2 (2, 6). The pancreatic metastasis and contralateral renal nodule had slightly decreased during treatment but were stable according to Response Evaluation Criteria in Solid Tumors (RECIST) 1.1 criteria. No new lesions or rapidly progressing lesions were observed.

**Table 1:** The thyroid neck group morbidity complexity score (adapted from references 5 and 8).

Score	Definition
0 (Mild)	- Disease confined to the thyroid and/or neck lymph nodes
1 (Moderate)	- Gross extrathyroidal and/or gross extranodal extension involving of muscle and/or internal jugular vein. - Esophageal muscularis or pharyngeal constrictor involvement
2 (Severe)	- Patients at risk for unilateral vocal cord paralysis: disease in one tracheoesophageal groove - Tracheal involvement requiring segmental tracheal resection < 4 cm - Superior mediastinal extension requiring sternotomy - Limited laryngeal cartilage resection - 11th and 12th nerve involvement
3 (Very Severe)	- Patients at significant risk for bilateral vocal cord paralysis: soft tissue disease in both tracheoesophageal grooves - Laryngopharyngeal and/or significant esophageal involvement (i.e., patient would require total laryngectomy or segmental esophageal resection) - Tracheal resection > 4 cm
4 (Unresectable)	- 360° carotid artery encasement, innominate artery encasement, prevertebral fascia involvement, and/or brachial plexus involvement

**Figure 3:**
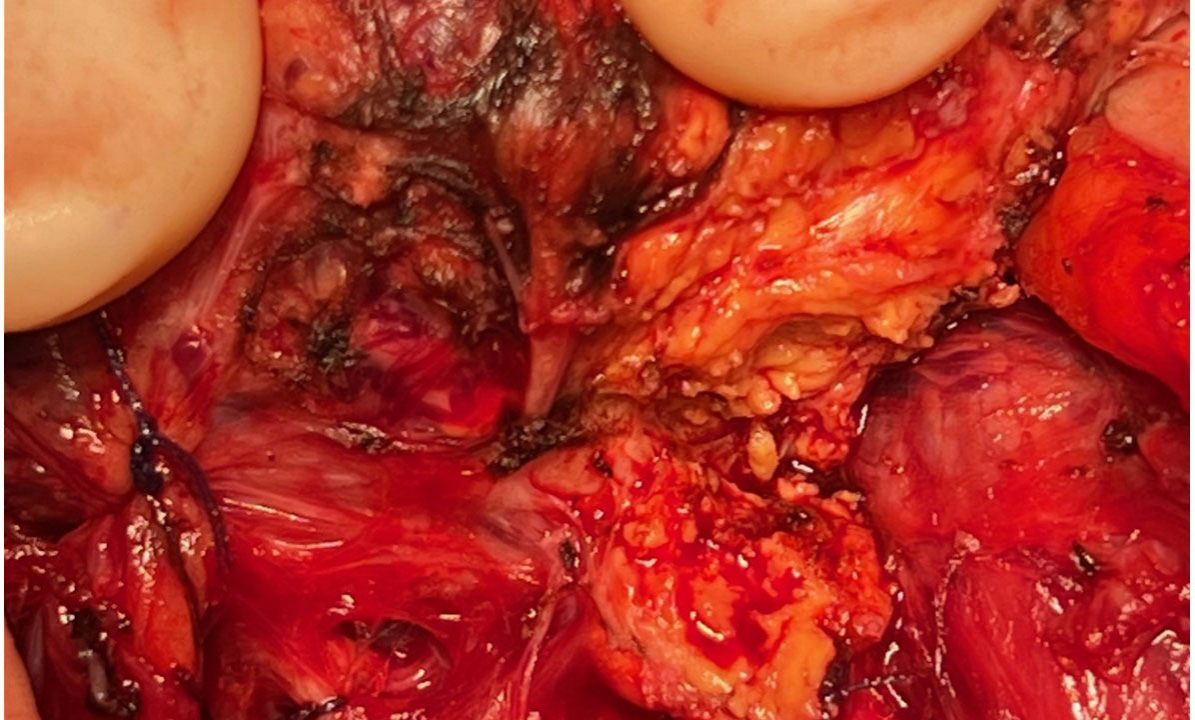
Recurrent nerve involvement by the tumor in patient 1.

**Figure 4:**
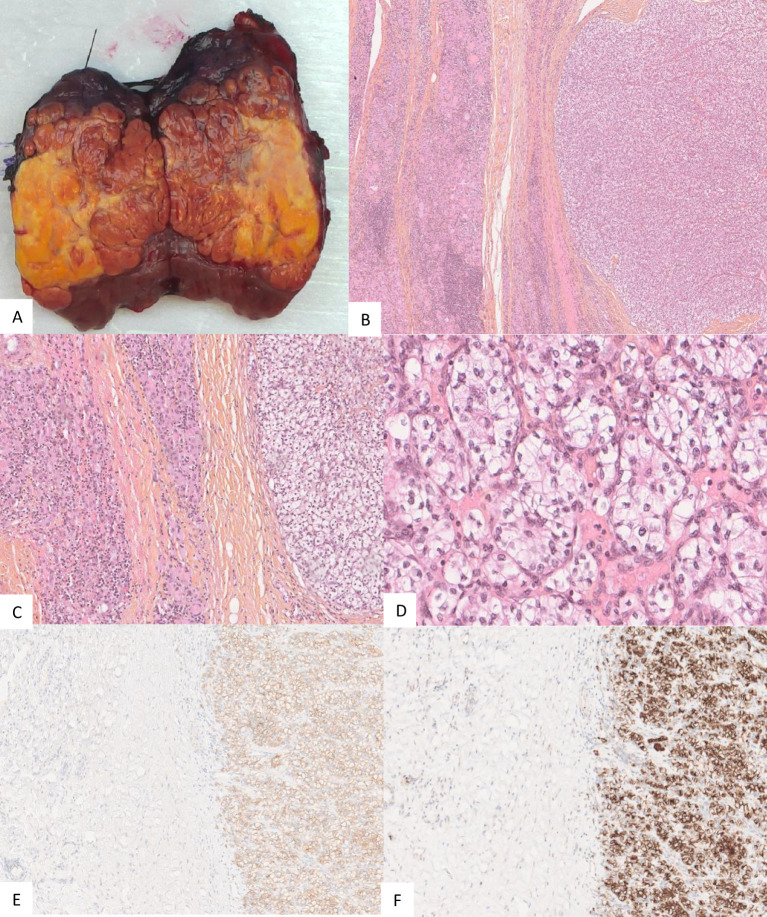
Gross appearance of the thyroid nodule for patient 1 with the yellowish part representing necrosis **(A)**. Pathology of the operating specimen showed normal thyroid tissue (left half of each image **(B, C)** abutting the ccRCC (right half of each image **(B, C)**. Tumor cells exhibited low atypia with an International Society of Urological Pathology (ISUP) nucleolar grade of 3 and clear cytoplasm **(D)**. Tumor cells expressed carbonic anhydrase IX (CA IX) **(E)** and cluster of differentiation 10 (CD10) **(F)**.

Considering her good performance status (Karnofsky performance score of 90%) and favorable-risk group according to the Memorial Sloan Kettering Cancer Center (MSKCC) and International Metastatic RCC Database Consortium (IMDC) prognostic models (9, 10) for RCC and the oligometastatic disease (less than five distant metastases), surgery was performed to relieve local symptoms of compression and decrease tumor burden. A total thyroidectomy was planned because of bilateral nodular disease, but only a partial lobectomy was performed on the right side because of loss of signal on recurrent nerve neuromonitoring during the dissection on the left side where the nerve was partially encased in residual tumor tissue (**Figure 3**). Resection was incomplete at the level of the nerve, leaving a small tumor remnant (R2). On pathology, a partial pathological response was noted with 80% residual viable tumor. Tumor necrosis and mature hyaline fibrosis, signifying partial tumor response, were observed (**Figure 4**).

The post-operative course was uneventful except for left vocal fold paralysis with dysphonia but without dysphagia. Hospital stay was 1 day. At 14 months postoperatively, systemic therapy had not been resumed and voice had completely recovered. Due to previous toxicity and the stability of the metastatic lesions, a new line of systemic therapy has not been proposed.

### Patient 2

**Figure 5:**
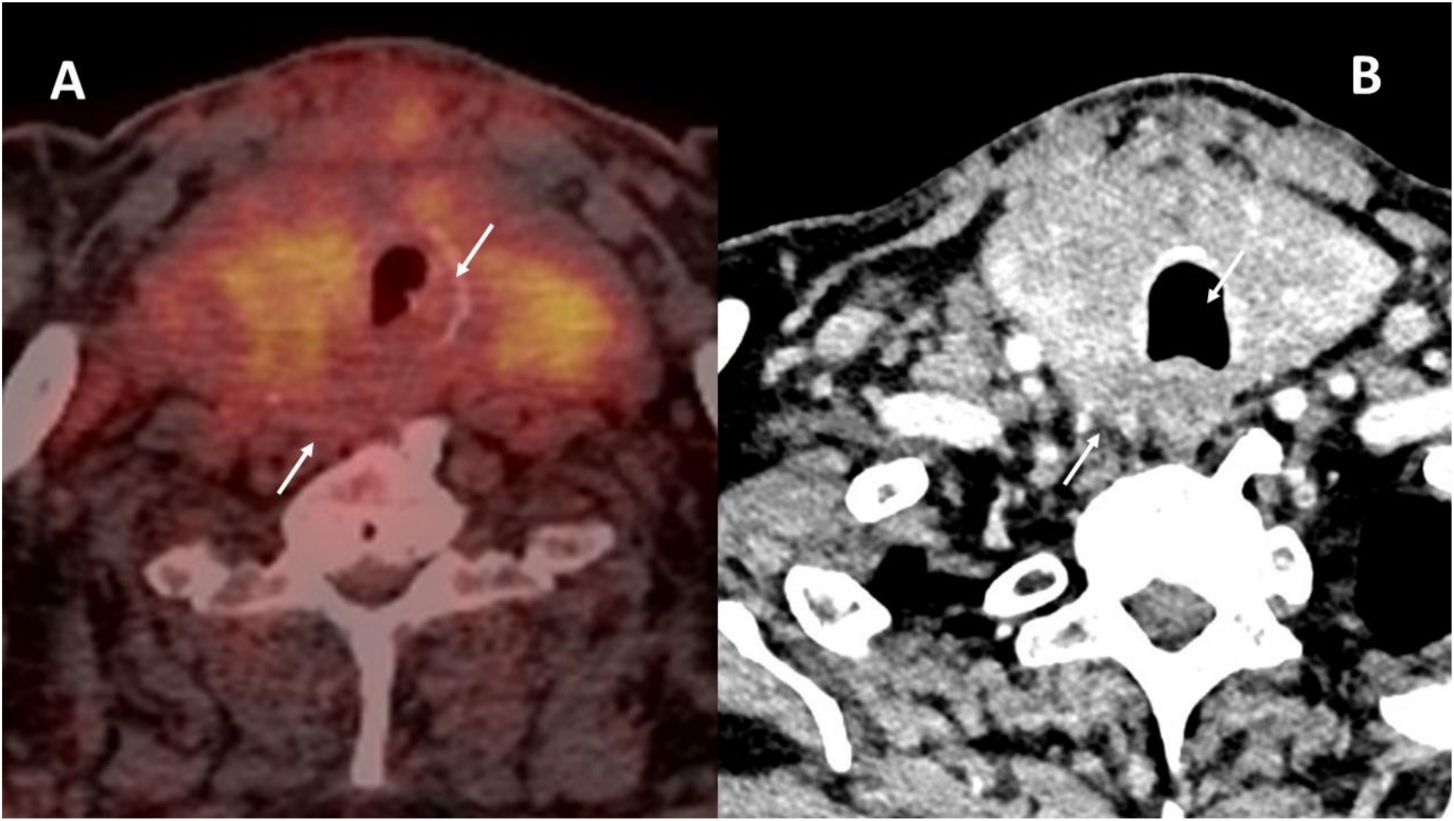
Tumor response in patient 2. Initial ^18^FDG-PET **(A)** as compared to the contrast-enhanced CT **(B)** after 4 months of cabozantinib and nivolumab. The initial tracheal invasion and involvement of the prevertebral muscles regressed with treatment (white arrows in both images).

The tumor in patient 2 showed a clinical response with a decrease in the volume of the thyroid mass and a decrease in the cranio-caudal extent of the thrombus in the right internal jugular vein. Grade I toxicity was observed: asthenia, muscle cramps, xerodermia, and intermittent diarrhea. In terms of the Thyroid Neck Group Morbidity Complexity Score (**Table 1**) (2, 6), the tumor regressed from class 4—unresectable—to class 1—moderate morbidity—internal jugular vein involvement. In terms of the MMM surgical morbidity complexity score, the lesion regressed from a score of 18 to a score of 2 (2, 6). The tracheal involvement completely disappeared as did the prevertebral fascia invasion (**Figure 5**). The thrombi persisted in both internal jugular veins but with blood flow reappearing in the left internal jugular vein.

**Figure 6:**
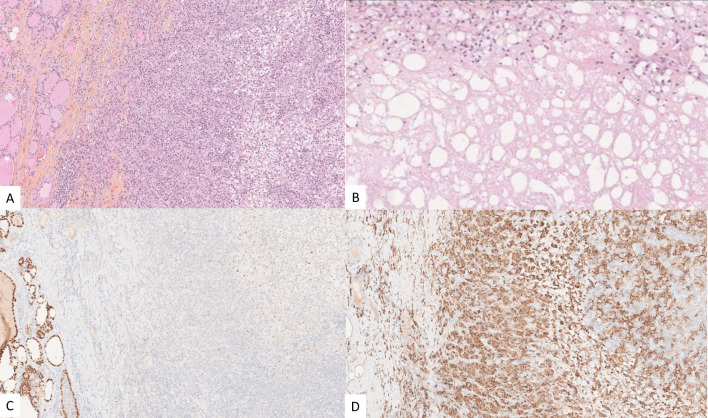
Post-therapeutic micrographs showing residual ccRCC (outlined in blue) and an important inflammatory reaction with a small focus of residual ccRCC **(A)**, tumor necrosis in the tumor bed **(B)**, PAX8 highlighting the residual viable tumor cells (outlined in blue) and the adjacent thyroid parenchyma **(C)**, and CD163 expressed by the foamy macrophages **(D)**.

Given the excellent response, the excellent performance status (Karnofsky score of 100%), the MSKCC and IMDC favorable-risk groups, and in the absence of other progressive distant metastases, the patient underwent total thyroidectomy and resection of the right internal jugular vein. Due to the volume of the thyroid metastasis, radiation therapy alone, requiring a high dose with a wide field, was considered at risk of being ineffective. Resection was macroscopically complete. On histopathological examination, a major pathological response was noted. Scattered residual viable tumor cells, estimated at 10%, were noted in the tumor bed. Signs of tumor regression included necrosis, fibrosis, and a dense macrophage infiltrate. The margins of the tumor were in fact only post-therapeutic fibrosis and necrosis (**Figure 6**).

The postoperative course was uneventful and hospital stay of 2 days. The patient underwent external beam radiation therapy to the neck to treat the residual tumor thrombosis in the left internal jugular vein, with no complications, and is currently at 6 months of follow-up with no progression of disease and no symptoms.

## Discussion

The advent of immunotherapy combined with kinase inhibitors has revolutionized the management of metastatic ccRCC, with an improvement in overall and progression-free survival in patients treated with nivolumab and cabozantinib versus sunitinib (11). In the CheckMate 9ER trial, the response rate with this combination was 55.7%, with 14% complete responders. Other combinations have also been shown to be effective, with 17% complete response rate combining lenvatinib and pembrolizumab (12). The use of these combinations in the preoperative or perioperative setting has not yet been shown to improve survival, but further clinical trials are underway (13).

Local therapies—surgery, radiation therapy, or other ablative techniques—when judged feasible by a multidisciplinary tumor board, are recommended for oligometastatic disease in selected patient (5, 14–16). Questions remain, however, as to the timing of local treatment or systemic therapy in oligometastatic patients because these lesions can remain stable over long periods of time (17, 18). Retrospective studies have shown a clinical benefit in highly selected patients who have undergone complete metastasectomy. This allows a drug holiday or a long delay before restarting systemic therapy in the event of disease recurrence (19). Favorable-risk patients according to the MSKCC and the IMDC risk criteria have been shown to benefit more from local therapy, as are patients achieving a complete resection of their metastases (19–21).

Thyroid metastases are rare, and there are limited, retrospective data concerning outcomes. Diagnosis of a thyroid nodule or mass found on imaging in a patient with a history of ccRCC relies on analysis of an ultrasound-guided fine-needle aspiration (FNA) biopsy, as for thyroid nodules in general (22). FNA should be analyzed with the suspicion of a metastasis and immunohistochemistry performed (23). In case of discrepancy, a core-needle biopsy with pathological analysis should be performed (3). The differential diagnosis for large, progressive thyroid masses includes anaplastic thyroid cancer, poorly differentiated thyroid cancer, and lymphoma, all of which require an emergency core needle biopsy for diagnosis and immunohistochemistry (24).

Beutner et al. performed a systematic review including 285 patients with metachronous thyroid metastases. The mean time to diagnosis was 8.8 years (25). Median survival after diagnosis of a thyroid metastasis was 3.4 years. In this review of the literature, there was no difference in survival between patients achieving complete resection in the thyroid versus incomplete resection. In the series of 45 patients published by Iesalnieks et al., 5-year overall survival was 51% in patients undergoing thyroid metastasectomy, with age ≥70 being a factor for poorer survival (26). Bader et al. reported one case of a patient treated with sunitinib prior to thyroid metastasectomy, and, to our knowledge, this is the only previously reported case of administering systemic therapy before performing a thyroidectomy for a thyroid metastasis from ccRCC (8). The authors reported an estimated 70% tumor response. Due to positive resection margins, adjuvant radiation therapy was administered.

Standard thyroid surgery in selected patients with thyroid metastases from ccRCC can provide local control in the neck with minimal morbidity, without preoperative systemic therapy (3). Thyroid lobectomy may be performed, but lesions have been reported to be bilateral and require a total thyroidectomy in 28%–57% of cases (4, 26–28). Extrathyroidal extension and invasion of the recurrent laryngeal nerve were factors for lower survival in the series of 17 patients reported by Machens et al. (28) In our two cases, the thyroid metastases were deemed unresectable due to invasion of functionally or vitally critical structures in the neck, with unacceptable morbidity or mortality expected from surgery. Systemic therapy with a tyrosine kinase inhibitor plus immunotherapy provided enough tumor response to perform surgery with low morbidity.

The RECIST 1.1 criteria used to evaluate tumor response do not accurately reflect resectability, which is determined by tumor invasion of functionally or vitally limiting structures in the neck (29).

With this consideration in mind, two classifications systems—the Thyroid Neck Group Morbidity Complexity Score and the MMM score (2, 6)—have been developed and are currently being evaluated for sensitivity and relevance in clinical trials involving locally invasive primary thyroid tumors (https://clinicaltrials.gov/study/NCT04321954, accessed 8 August 2024). The aim is to provide an objective metric to measure tumor response and resectability in these complex tumors. The Thyroid Neck Group Morbidity Complexity Score is comprised of five levels of increasing complexity from 0 to 4 (**Table 1**). Unresectable tumors (level 4) show 360° encasement of the carotid or innominate arteries, prevertebral fascia involvement, and/or brachial plexus involvement. Similarly, the MMM score increases as the complexity and morbidity of a potential surgical resection increases. A score is attributed to each functionally or vitally limiting structure, with the final score being the sum of the composite scores. A score of 9 is attributed each to carotid artery or innominate artery encasement, prevertebral fascia involvement, and brachial plexus involvement, whereas a unilateral vagus or recurrent nerve involvement is scored with 2, and a score of 1 is attributed to the internal jugular vein or strap muscles. Response may also be described by clinical features—tumor mass, fixation on palpation, or regression of recurrent nerve paralysis (6)—but the critical structures involved are the key to evaluating resectability.

In our two cases, the Thyroid Neck Group Morbidity Complexity Score was 4 preoperatively and 2 and 1, respectively, after systemic therapy. The response with the tumor “retreating” from the functionally critical structures was reflected in the change of score, as well as in the change in the MMM score from 18 to 2 in both patients. It is to note that the term “neoadjuvant” applies generally to systemic therapy administered for tumors deemed unresectable at the primary renal site, so “preoperative systemic therapy” may be a more appropriate term for metastatic disease sites such as the thyroid that are subsequently treated surgically.

The criteria for assessing pathological response following immunotherapy in metastatic ccRCC involve a range of histological changes indicative of tumor regression that provides insight into treatment efficacy. Central to this histological evaluation is the presence of tumor necrosis, which is a hallmark of effective immunotherapy and anti-angiogenic tyrosine kinase inhibitors.

Additionally, assessing the extent of viable tumor cells, alongside immune cell infiltration within the tumor microenvironment plays a crucial role. Changes in the surrounding stroma, such as fibrosis, cholesterol clefts, and foamy histiocytes, provide additional insights into tumor response (30, 31). A complete pathological response is characterized by the absence of viable tumor cells, whereas partial response may indicate significant necrosis or reduced tumor burden alongside a notable immune response. However, even in cases with a complete pathological response and no viable tumor cells, scar tissue, fibrosis, and necrosis may still persist, making radiological evaluation difficult (32).

Despite the tumor response leading to a functionally resectable tumor, tumor margins in patient 1 were R2 in an attempt to preserve left recurrent nerve but were R0 at the level of the thyroid in patient 2, due to the almost complete pathologic response observed (**Figure 6**). The degree of response in patient 1 was possibly due to the shorter treatment period. Furthermore, pathologic response and morphological response on imaging are often discordant after systemic therapy with immunotherapy (33). Surgical margins are never wide in these cases, due to the proximity of the tumor to the trachea, larynx, esophagus, great vessels, and cranial nerves. Thus, adjuvant radiation therapy or continuation of systemic therapy should be discussed in a multidisciplinary setting.

The risk of permanent morbidity after thyroid surgery should be weighed against the tolerance and toxicity of systemic therapy in cases of progressive and/or symptomatic thyroid metastases from ccRCC, with a discussion in a multidisciplinary setting (15, 16, 34). In the two cases presented here, surgery without prior neoadjuvant systemic therapy was judged to be too morbid, due to the structures involved by the tumor. After thyroid surgery, patients were able to discontinue systemic therapy due to stability in the other metastases. Thus, thyroid surgery had the advantage of treating the dominant metastasis with low morbidity while limiting the duration of systemic therapy, at least until a new progression is observed.

## Conclusions

Thyroid metastases from ccRCC are generally amenable to classic thyroid surgery, when indicated for oligometastatic patients, after discussion in a multidisciplinary tumor board setting, according to current recommendations. In the two cases presented, the thyroid metastases were the predominant and progressive lesions in otherwise oligometastatic patients in a favorable IMDC group but were unresectable due to involvement of critical anatomic structures in the neck. Systemic therapy with a combination of kinase inhibitor and immunotherapy led to tumor regression to the point where the thyroid tumor became resectable with low morbidity. This paradigm may be useful in selected patients with these rare thyroid metastases, but the impact on overall survival remains to be determined.

## Data Availability

The original contributions presented in the study are included in the article/supplementary material. Further inquiries can be directed to the corresponding author.
